# Design and synthesis study of the thermo-sensitive poly (N-vinylpyrrolidone-b- N, N-diethylacrylamide)

**DOI:** 10.1080/15685551.2018.1448230

**Published:** 2018-03-13

**Authors:** Xiayun Zhang, Zhongduo Yang, Dengmin Xie, Donglei Liu, Zhenbin Chen, Ke Li, Zhizhong Li, Brandon Tichnell, Zhen Liu

**Affiliations:** a College of Life and Engineering, Lanzhou University of Technology, Lanzhou, China; b State Key Laboratory of Gansu Advanced Non-ferrous Metal Materials, Lanzhou University of Technology, Materials Science and Engineering, Lanzhou, China; c Department of Physics and Engineering, Frostburg State University, Frostburg, MD, USA

**Keywords:** RAFT polymerization, block copolymer, thermo-sensitivity, synthesis, characterization

## Abstract

The reversible addition fragmentation chain transfer (RAFT) polymerization method was adopted here to prepare a series of thermo-sensitive copolymers, poly (N,N-diethyl- acrylamide-b-N-vinylpyrrolidone). Their structures, molecular weight distribution and temperature sensitivity performances were characterized by the nuclear magnetic resonance (^1^HNMR), the gel permeation chromatography (GPC) and the fluorescence spectrophotometer, respectively. It has been identified that the synthesis reaction of the block copolymer was living polymerization. The thermo-sensitivity study suggested that N-vinylpyrrolidone (NVP), played a key role on the lower critical solution temperature (LCST) performance.

## 1. Introduction

Block copolymers, also known as mosaic copolymer, is a special polymer which linked two or more polymer segments on the main chain directly [1]. Besides containing related properties of each block polymer, block copolymers also provide some novel properties, such as self-assembly, micro-phase separation, et al. [2–5], and which make them show an extensive application prospect. Among block copolymers, the thermosensitive block copolymer has been developed rapidly in recent years because the thermosensitive segment enabled the sol/gel phase transfer in selective solvents at their special LCST (Specifically, as temperature is higher than LCST, polymer would gather together and separate from solvent, while it would dissolve to the solvent again reversibly as temperature is lower than LCST). What’s more, during the gelatin process, thermo-sensitive block polymers will self-assemble and form a structure with a hydrophobic core-hydrophilic shell, which have shown a broad application as a prospect in electronic engineering [6, 7], drug release [8, 9], separation [10], nanomaterials [11], tissue engineering [12, 13], and so on. Reports on poly (N-isopropylacrylamide) (PNIPAM) block copolymer have been carried out more systematic and in-depth [14], but the poor biocompatibility and low LCST have seriously affected its future application prospects in medicine fields.

Compared with PNIPAM, a later found out thermo-sensitive polymer, poly (N, N-diethylacrylamide) (PDEA), has shown better biocompatible [15] property and higher LCST [16], which make the application of thermo-sensitive in polymer drug become more practical. Just for this reason, study about PDEA has attracted widely attention all over the world, and many reports about it could be indexed [17–21]. In modification aspect, researchers are mainly focused on improving LCST of PDEA or appending some other special properties to make it more versatile in future application [22–24]. However, multifunctional modifications were mainly realized by random copolymerization [25–27], the method about block copolymer is relatively small [21, 28–33], and the block copolymer of poly (N, N-diethylacrylamide-b-N-vinylpyrrolidone) was even not indexed at present. According to report, poly N-vinylpyrrolidone (PVP) is a kind of non-ionic water-soluble polymer, which possesses desirable properties such as biocompatibility, film-forming ability, biodegradability, and so on. Therefore, the property of block copolymer containing PVP and other segments has also been reported extensively [34–39]. Based on the nearer LCST to body temperature of PDEA and the excellent biocompatibility of PVP, it could be deduced that if combining PDEA together with PVP by block polymerization, and if LCST of block copolymer increased to equal to the body temperature, the supper properties of this block copolymer would show a very promising prospect in pharmaceutical field.

Generally, a block copolymer with a well-designed structure is usually prepared by the living radical polymerization method because the living radical polymerization always exists a reactive end groups and can be used to polymerize different monomers. For example, common monomers have styrene, methacrylates, acrylonitrile, vinyl acetate, etc., functional monomers have acrylic acid, sodium styrene sulfonate, dimethylaminoethyl methacrylate, etc. [40]. What’s more, polymer prepared by the living polymerization possessed a relative definite molecular weight and narrow molecular weight distribution [41], which would facilitate the study about properties of polymers with different structures in detail. According to reports, living radical polymerization methods always adopted contain nitroxide-mediated radical polymerization (NMP) [42], atom transfer radical polymerization (ATRP) [43] and RAFT [44] etc. Among them, RAFT is more popular since it can control the polymerization process better for both non polar and polar monomers. Besides, the monomer can be selected widely, the operation condition is more mild and assessable, the monomer conversion rate of RAFT is higher [45, 46], and it can be well applied in polymerization system of solution, emulsion and suspension [47–51], At the moment, the composition, sequential structure and molecular weight of block copolymer prepared by RAFT can be understood and calculated, and the end-group can be changed to others using an easy and mild chemical reaction because the existence of thiol end group [52]. At present, RAFT has been used extensively for the synthesis of the multi-block copolymer which requires multiple isolations and dispersion of the polymer [53, 54].

Based on the above findings, the preparation of DV using RAFT method was carried out in this work, the process was completed as follows: DV was prepared by polymerized PDEA, DV, in turn, using RAFT polymerization technology, and the structure contained the content of each block, the molecule weight and polydispersity index(*PDI*) were characterized by ^1^HNMR and GPC, respectively. All results documented that the polymerization was living polymerization. Then, the thermo-sensitive copolymer of DV was characterized, and the result of thermo-sensitivity about NVP showed that increasing the molar ratio of NVP, LCST increased, while the thermo-sensitivity was weakened, and when the ratio of n_DEA_:n_NVP_ was in the range of (1:0.04, 1:0.05), LCST approximated to 36 °C, which was very close to body temperature.

## 2. Experimental

### 2.1. Materials

Dodecylmercaptan (AR), Sodium hydride(AR), Carbon disulfide (AR), Iodine (AR), Sodium thiosulfate (AR),and Sodium sulfate (AR) were provided by the YantaiShuangshuang Chemical Co. Ltd. Ethyl acetate (AR), Petroleum ether (AR), Ether (AR),n-hexane (AR),and n-decane(AR) were purchased by the Rionlon Bo hua pharmaceutical chemical company. Acenaphthylene(AR) was provided by the Shanghai SA Ren Limited Liability Company. Acetone (AR) was produced by Beijing Chemical Works. All above reagents were used directly. Azobisisobutyronitrile(AR) was provided by YantaiShuangshuang Chemical Co. Ltd and was recrystallizedby95% ethanol before using.

### 2.2. Preparation of trithiocododecanoic acid-2-cyanoisopropyl

**Scheme 1.**
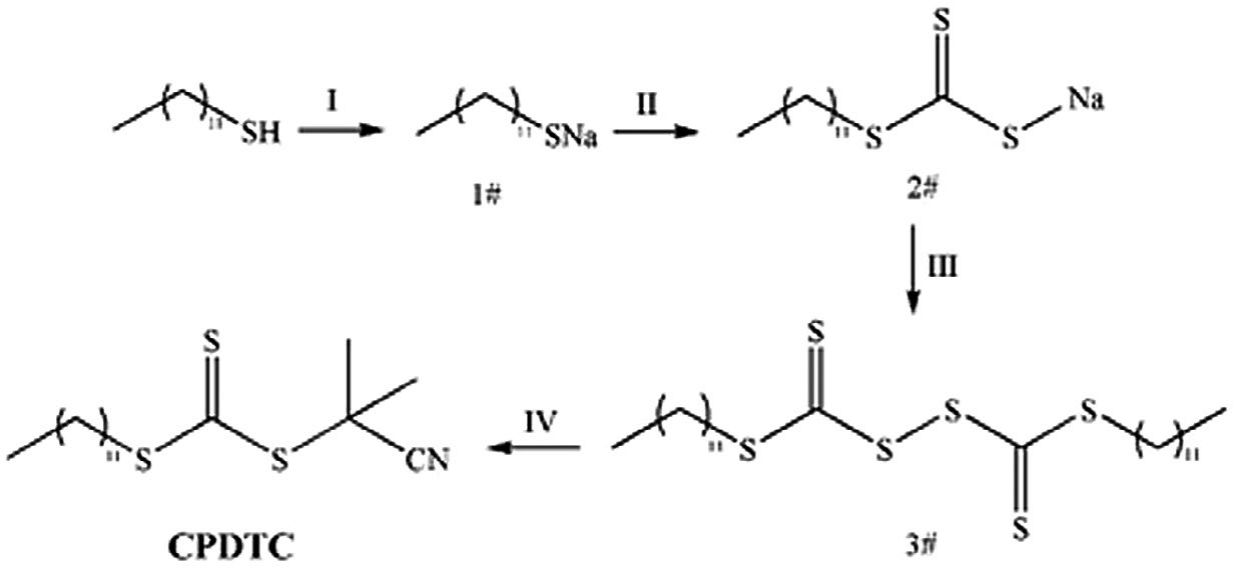
The mechanism of CPDTC synthesis process.

**Scheme 2.**
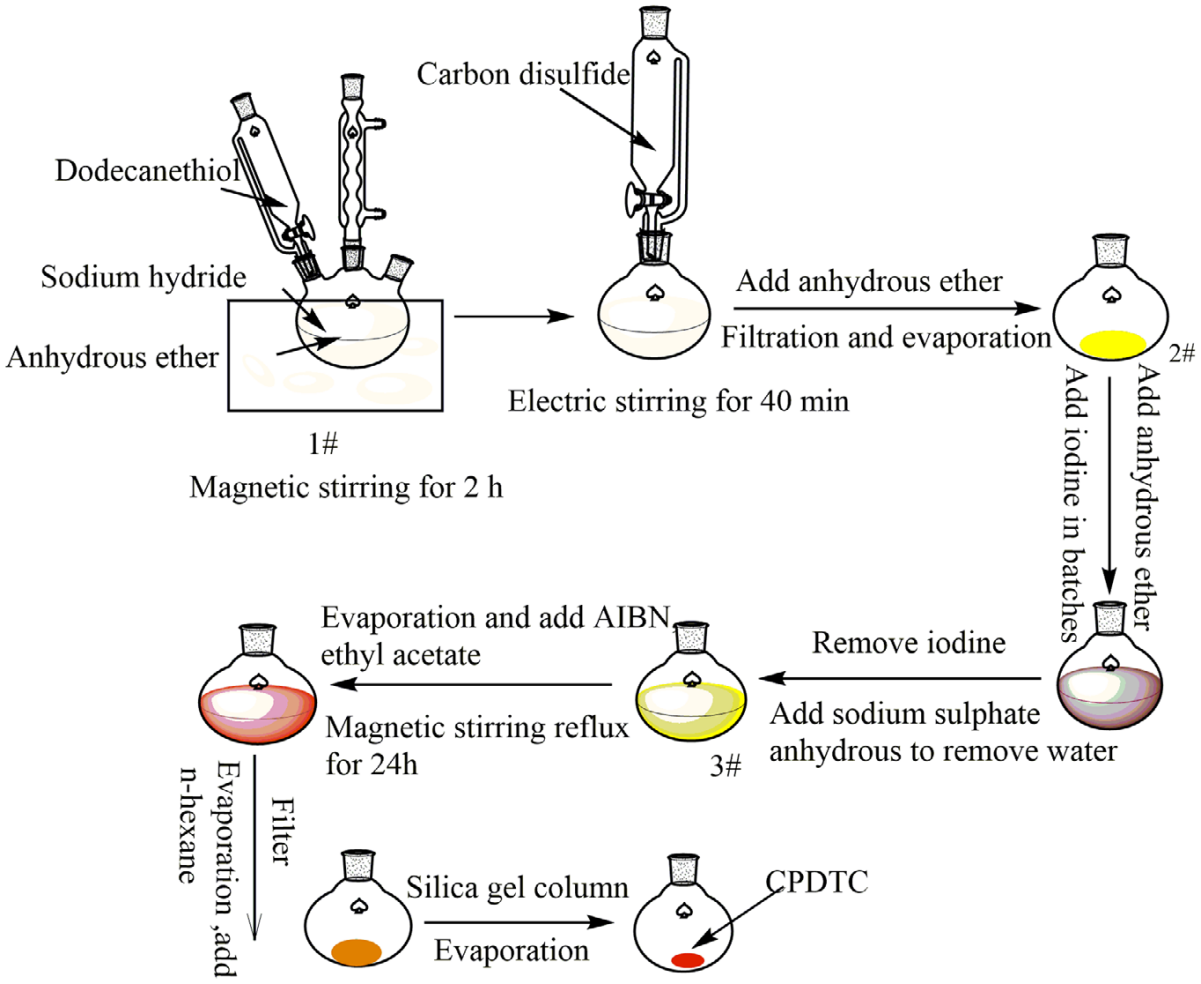
CPDTC synthesis process.

The synthesis theory of trithiocododecanoicacid-2-cyanoisopropyl (CPDTC), a RAFT agent, was shown in Scheme 1, and the process was shown in Scheme 2. A solution of sodium hydride in anhydrous diethyl ether (1.58 g sodium hydride was dissolved into 75 mL anhydrous ether) was loaded into a 250 mL three necked flask which was assembled with a dropping funnel and a thermometer. Then the flask was fixed on a magnetic stirrer equipped with an ice-water bath. After the solution (1#) was agitated and cooled to room temperature, carbon dodecylmercaptan (7.7 g) was added to the system with the rate of one drop for each two seconds (1d/2s). After all carbon dodecylmercaptan was transferred to the reaction system, the reaction was kept for another 2 h under the ice-water environment. After that, the agitation system was changed with an electric stirrer, and carbon disulfide (3 g) was added dropwise slowly to the system, the all dripping time was controlled in 10 min. Keeping the system under electric stirring and reaction for 40 min, when the reaction was finished, the obtained crude product was dissolved in ether and separated the white block insoluble matter (dodecyl-mercaptan) by filtration, the filtrate was concentrated to a yellow viscous product (2#). After dissolving, the yellow viscous product in the ether under a magnetic stirrer, iodine (5.8 g) was added into the solution in batches. After reacting for 1 hat room temperature, sodium iodide was filtered off, and a dark brown filtrate was obtained. After the iodine in the system was removed by adding the aqueous solution of sodium thiosulfate, the mixture was separated by a separating funnel, and the oil phase was dried with anhydrous sodium sulfate until no block appeared as some sodium sulfate was added. When the filtration was stopped, the filtrate (3#) was evaporated till ether was removed completely. Fixing the system on an oil bath at 79 °C, AIBN (3.4 g) and ethyl acetate (80 mL) were added in turn. Keeping the reaction for 24 h under magnetic stirrer and reflux state, the mixture was concentrated and filtrated again. After that, n-hexane was added to the filtrate till no white needle-like precipitate was produced. Filtering the mixture and evaporating the filtrate again, the crude product of CPDTC was obtained. Finally, the crude product of CPDTC was refined by a silica gel column. The process was conducted as follows: silica gel (140 g) was dispersed into petroleum ether (500 mL), then the dispersion was injected into a silica gel column. Adjusting the silica gel column till the upper surface of silica was flat, and keeping the petroleum ether flowing until it reached a distance of about 0.5 cm from the upper surface of the silica gel, the piston was closed and the crude product of CPDTC was injected into the silica gel column with a pipette. After that, the piston was opened again. Introducing petroleum ether to the silica gel column persistently until the entire color belt diffused to the middle of the silica gel column and was washed off, the silica gel column was washed off with a mixture of petroleum ether and ethyl acetate (the volume ratio of petroleum ether and ethyl acetate was 12: 1) until the belt that existed on the upper surface of the silica gel column was washed completely. Collecting and evaporating the elution of petroleum ether and ethyl acetate, pure CPDTC with 99% was obtained.

### 2.3. Preparation of N, N- diethylacrylamide

N, N- diethylacrylamide and its precursor, acryloyl chloride, were prepared according to literature [55], and the product was characterized by^1^HNMR.

### 2.4. Preparation of block copolymer

#### 2.4.1. Preparation of PDEA

**Scheme 3.**
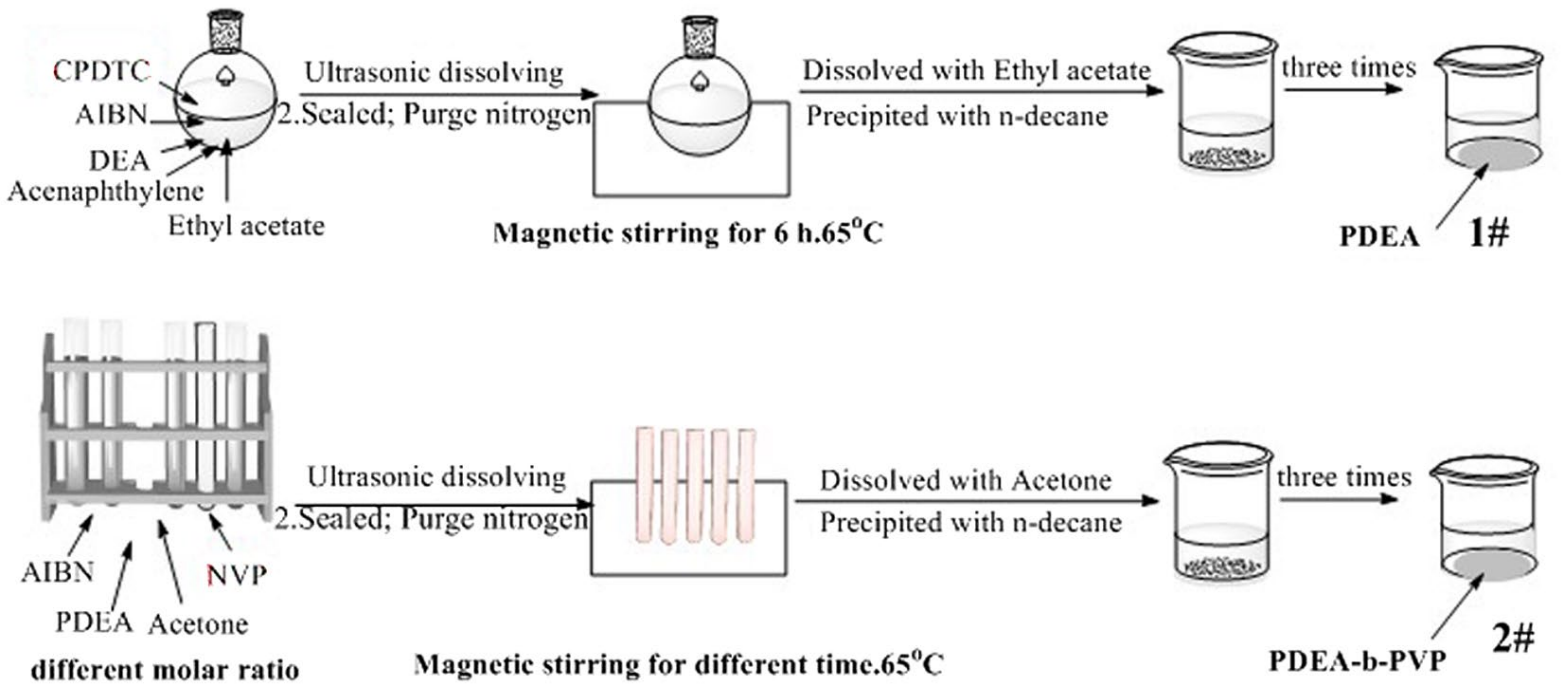
Synthesis of block copolymer DV.

Synthesis of PDEA was shown in Scheme 3, the product was 3# of Scheme 3: A solution of ethyl acetate (24 g), acenaphthylene (0.0661 g) and DEA (24 g) was added into a three necked flask, then CPDTC and AIBN were added in turn with the molar ratio of AIBN: CPDTC: DEA = 1: 5: 1000, and the mass ratio of DEA to acenaphthylene was 1.2700: 0.0035. After a nitrogen purge for 30 min, the flask was placed on an oil bath of 70 °C. Keeping the reaction under a persistent nitrogen purge and stirring for 24 h, the reaction was stopped, and the product solution was diluted with about 3 times the volume of ethyl acetate. After introduced, the solution dropwise slowly to about 10 times volume of n-decane under quickly stirring, the mixture was filtrated. Repeating the dissolution and precipitation process for three times, the final obtained precipitate was dried in a 40 °C vacuum oven under the vacuum degree of 0.08 to constant, and PDEA was obtained.

#### 2.4.2. Preparation of DV

Synthesis of DV was shown in Scheme 3, the product was 4# of Scheme 3. The synthesis process of DV was similar to that of PDEA except for PDEA here was conscripted as a RAFT reagent, NVP was used as a monomer, and the solvent was changed to acetone, the reaction time was different. After the final obtained precipitate was dried in a 40 °C vacuum oven under the vacuum degree of 0.08 to constant, different molar ratios of DV were obtained.

### 2.5. Structure characterization of RAFT reagents, monomers and polymers

Deuterated chloroform (CDCl_3_) solution (10 mg/mL) of CPDTC, PDEA and DV were prepared, respectively. And then^1^HNMR for each compound was determined by nuclear magnetic resonance spectroscopy (AV-400, Bruker, U.S.A.), and the molecular structure was characterized according to the chemical shift of hydrogen in each compound.

### 2.6. Characterization of molecular weight distribution

Tetrahydrofuran (THF, 5 mg/mL) solution of PDEA and DV were prepared, respectively, and the molecular weight distribution for each compound was detected by a gel permeation chromatograph (GPC, Waters 1525/2414/2487, Fairburn industrial development co. LTD, Shanghai, China) which was calibrated with narrowly distributed polymethyl methacrylate. During this process, DMF in which 0.05 mmol/L lithium bromide was contained was adopted as the mobile phase, and the injection volume was 10 μL at 1 mL/min flow rate and 25 °C.

### 2.7. Characterization of thermo-sensitive performance of copolymer

The thermo-sensitive performance of DV was determined by static fluorescence spectroscopy technology using a fluorescence spectrometer (LS-55, Perkin-Elmer Cetus Corporation, USA). During this process, 0.01 mg/mL of DV aqueous solution was prepared, and operation conditions were set as follows: the excitation wavelength was 290 nm, the scanning speed was 240 nm/min, the excitation and emission slit were10 nm, the scanning emission wavelength was 330–430 nm, and the fluorescent intensity determination wavelength was 380 nm.

## 3. Results and discussion

### 3.1. Structure of monomer and polymer

#### 3.1.1. Structure of CPDTC

**Figure 1.**
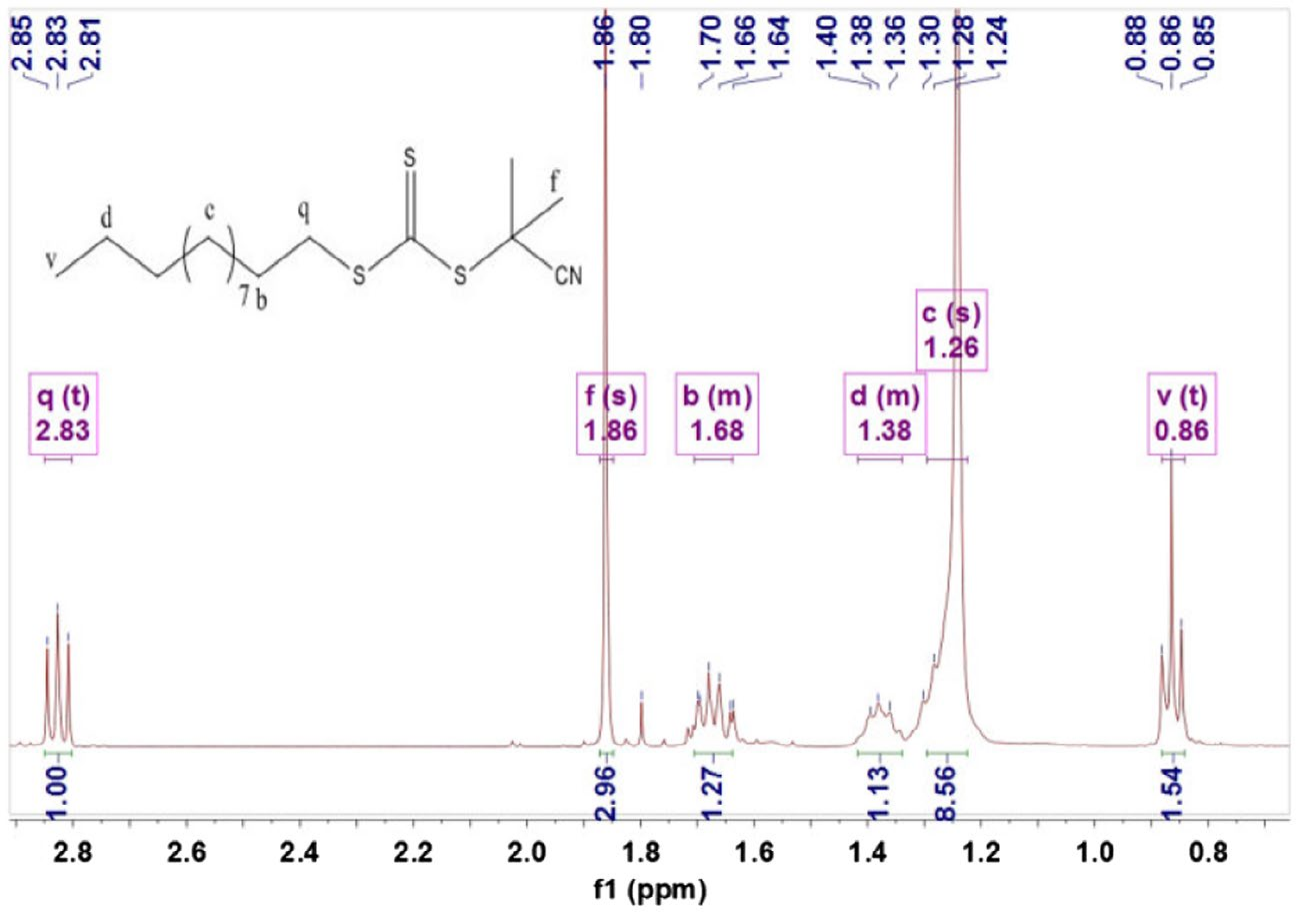
^1^HNMR spectra of the CPDTC.

Figure 1 shows ^1^HNMR (400 MHz, CDCl3, *δ*, ppm) spectra of CPDTC: 2.84 (t, 2H, –CH2–), 1.88 (s, 6H, –CH3), 1.72–1.65 (m, 2H, –CH2–), 1.26 (s, 16H, –CH2–) and 0.87 (t, 3H, –CH3).

#### 3.1.2. Structure of DEA and NVP

**Figure 2.**
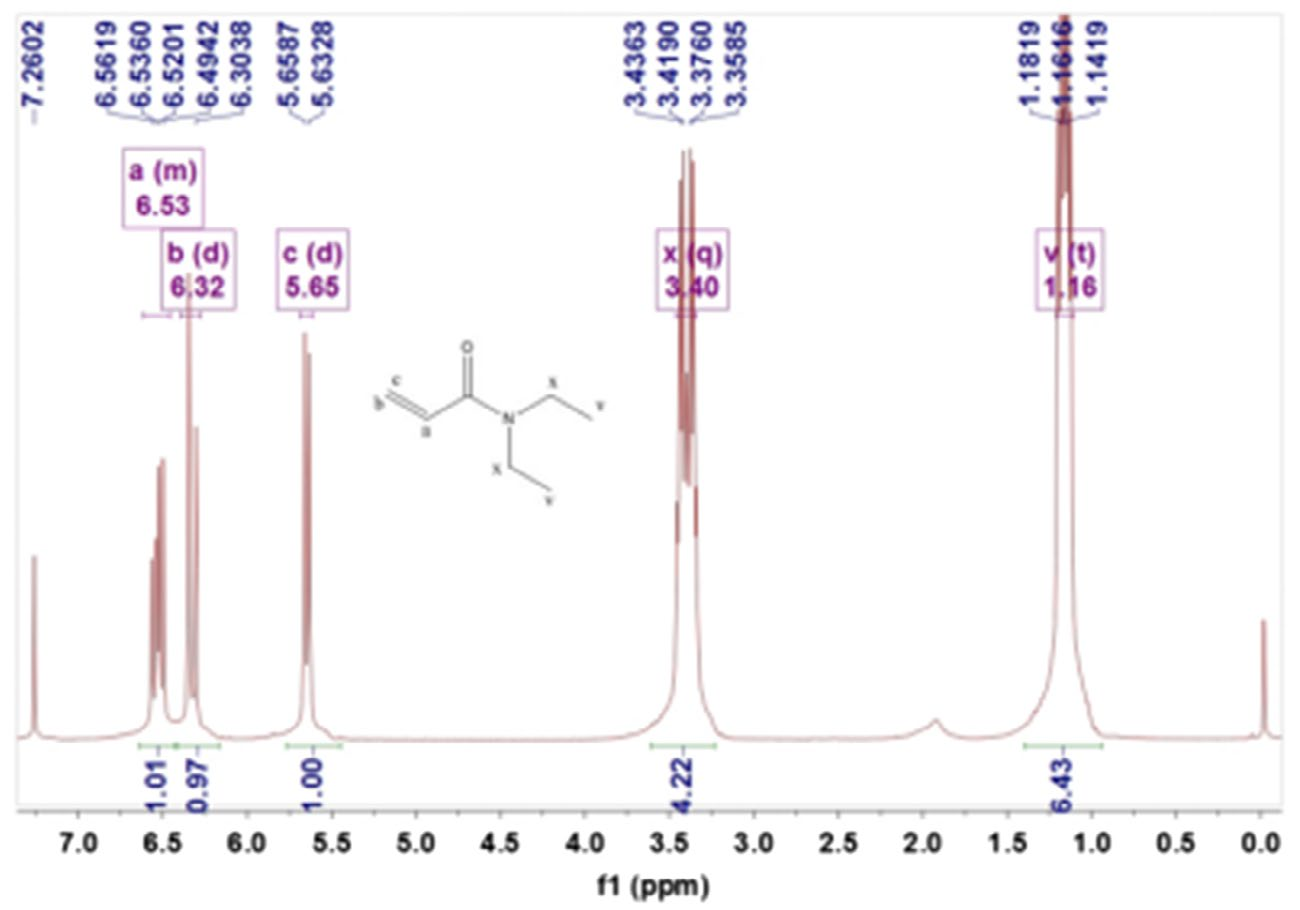
^1^HNMR spectra of the DEA.

Figure 2 showed ^1^HNMR (400 MHz, CDCl3, *δ*, ppm) spectra of DEA:1.16 (t, 6H, –CH3), 3.40 (q, 4H, –CH2–), 5.65 (dd, 1H, CH2=), 6.32 (dd, 1H, CH2=), 6.53 (m, 1H, CH2=CH–).

**Figure 3.**
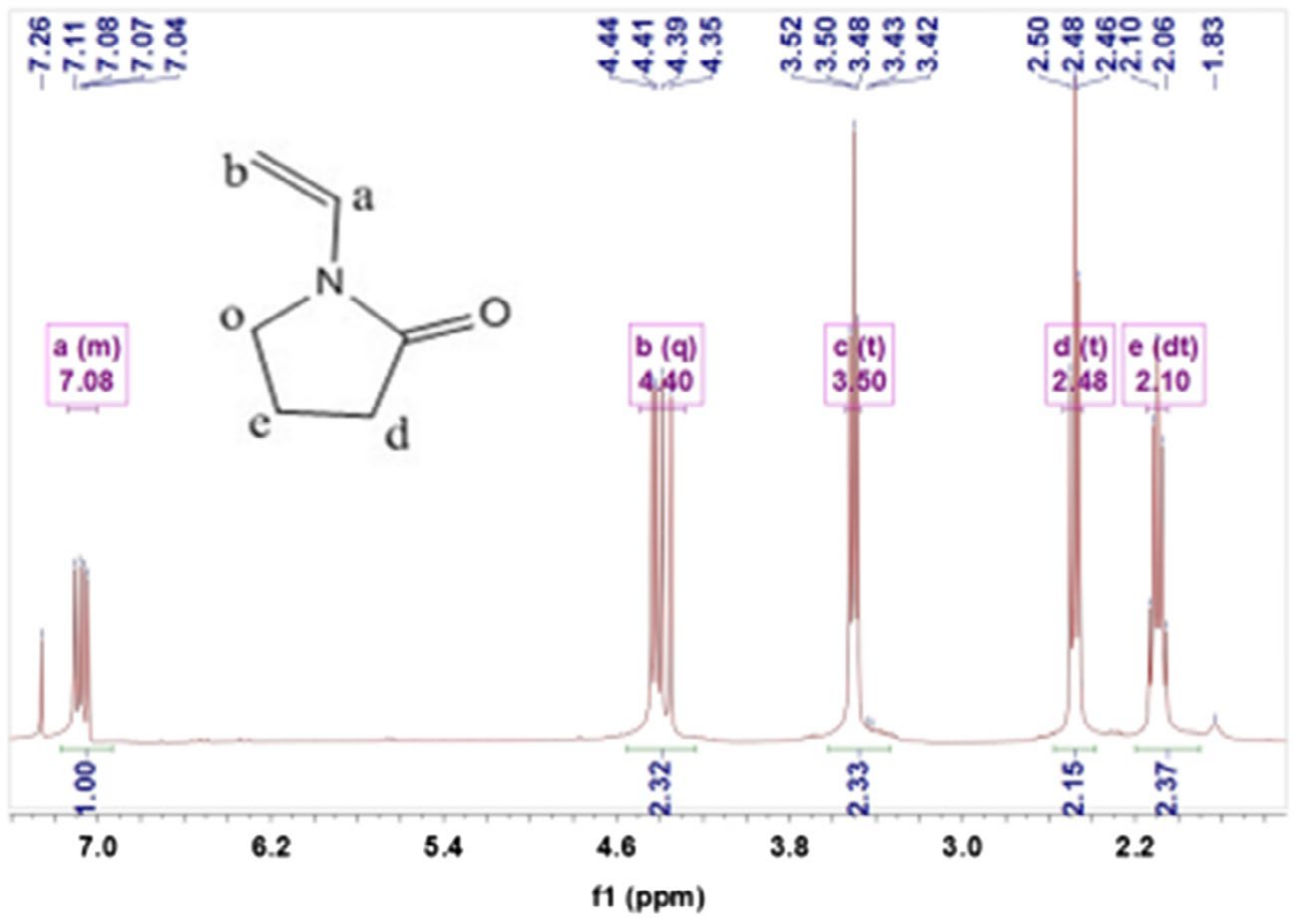
^1^HNMR spectra of NVP.

Figure 3 showed ^1^HNMR (400 MHz, CDCl3, *δ*, ppm) spectra of NVP:2.10 (dt, 2H, –CH2–), 2.48 (t, 2H, –CH2–), 3.50 (t, 2H, –CH2–), 4.40 (q, 1H, CH2=), 7.08 (m, 1H, CH2=CH–)

#### 3.1.3. Structure of PDEA

**Figure 4.**
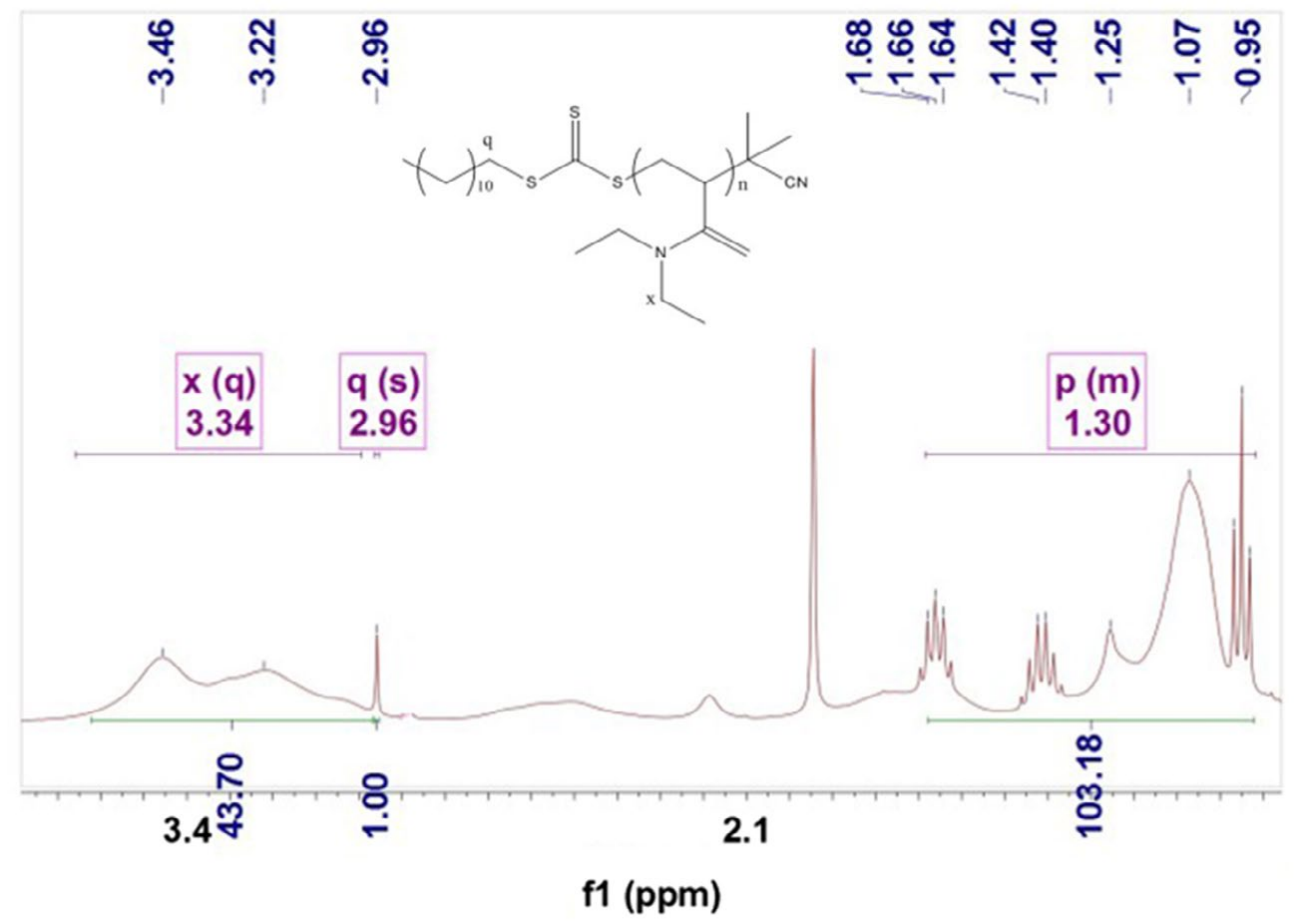
^1^HNMR spectra of the PDEA.

Figure 4 shows the ^1^HNMR spectra of PDEA. It could be found in a polymer, except for peak at *δ* = 2.96 in CPDTC and *δ* = 3.34 in DEA, almost all peaks for both CPDTC and DEA were submersed and could not be identified. The reason could be ascribed to large molecule of PDEA, because of the polymerization, large amounts of DEA were bonded together, and the quantities of hydrogen for a given group would increase hundreds of times. Due to the superposition of each group, and the influence of other groups adjacent to them both in link and in space, peaks related to a given group could not be distinguished clearly, naturally. However, due to the far *δ* distance of q and x from the others, the influence they suffered was relatively small, and could be conscripted as reference to calculate molecular weight of PDEA as Equation (1):

(1) $${\overset{¯}{M}}_{n}\left(\text{PDEA}\right)=\frac{\int \text{H}\left(\delta =3.34\right)}{\int 2\text{H}\left(\delta =2.96\right)}\times 127$$

where, $\int 2\text{H}\left(\delta =2.96\right)$ and $\int \text{H}\left(\delta =3.34\right)$ represent the total hydrogen quantities of hydrogen at chemical shifts 2.96 and 3.34, respectively. There are 2 mol H at *δ*(2.96) in 1 mol CPDTC, but 4 mol H at *δ*(3.34) in DEA, so as 1 mol CPDTC was combined with 1 mol DEA, the integral of H at *δ*(3.34)would be 2 times that at *δ*(2.96), 127 was the molecular weight of DEA.

For example, from Figure 3, it could be found the peak area ratio of q and x was 1: 43.70, so the molecular weight of PDEA should be 43.70/2 × 127 = 2774.95 g/mol (±0.2%).

Besides, GPC characterization showed a polydispersity index (*PDI*) of PDEA as 1.05, which was far smaller than the general free radical polymerization, and it indicated that the process of preparing PDEA was living polymerization.

#### 3.1.4 Structure of DV

**Figure 5.**
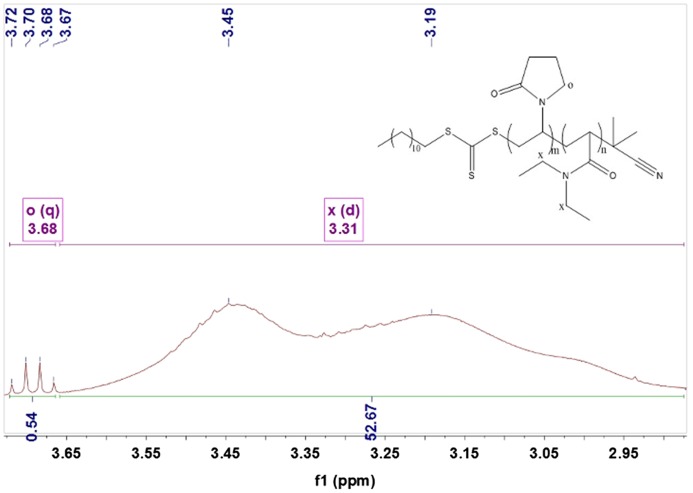
^1^HNMR spectra of the DV1.

**Figure 6.**
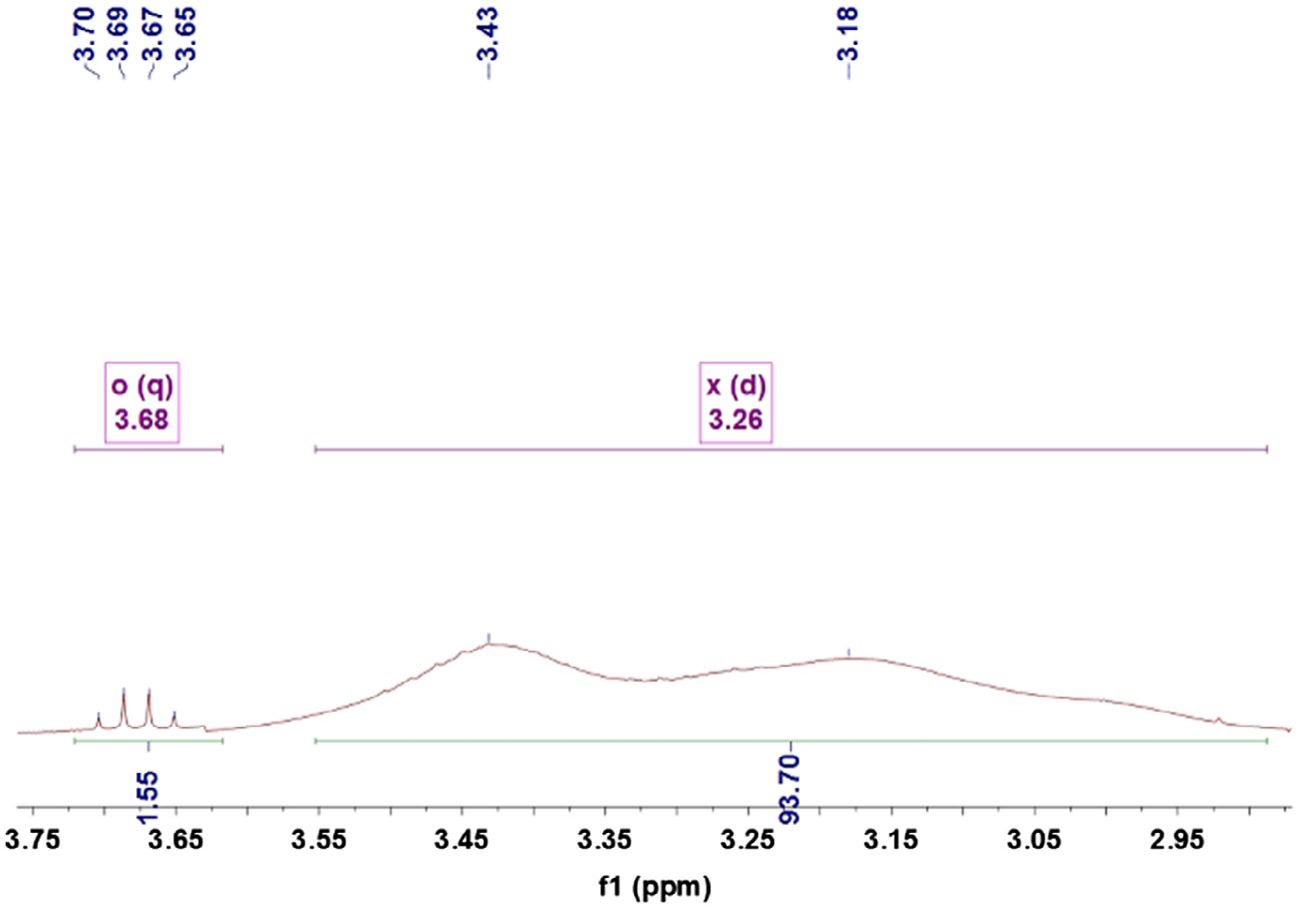
^1^HNMR spectra of the DV2.

**Figure 7.**
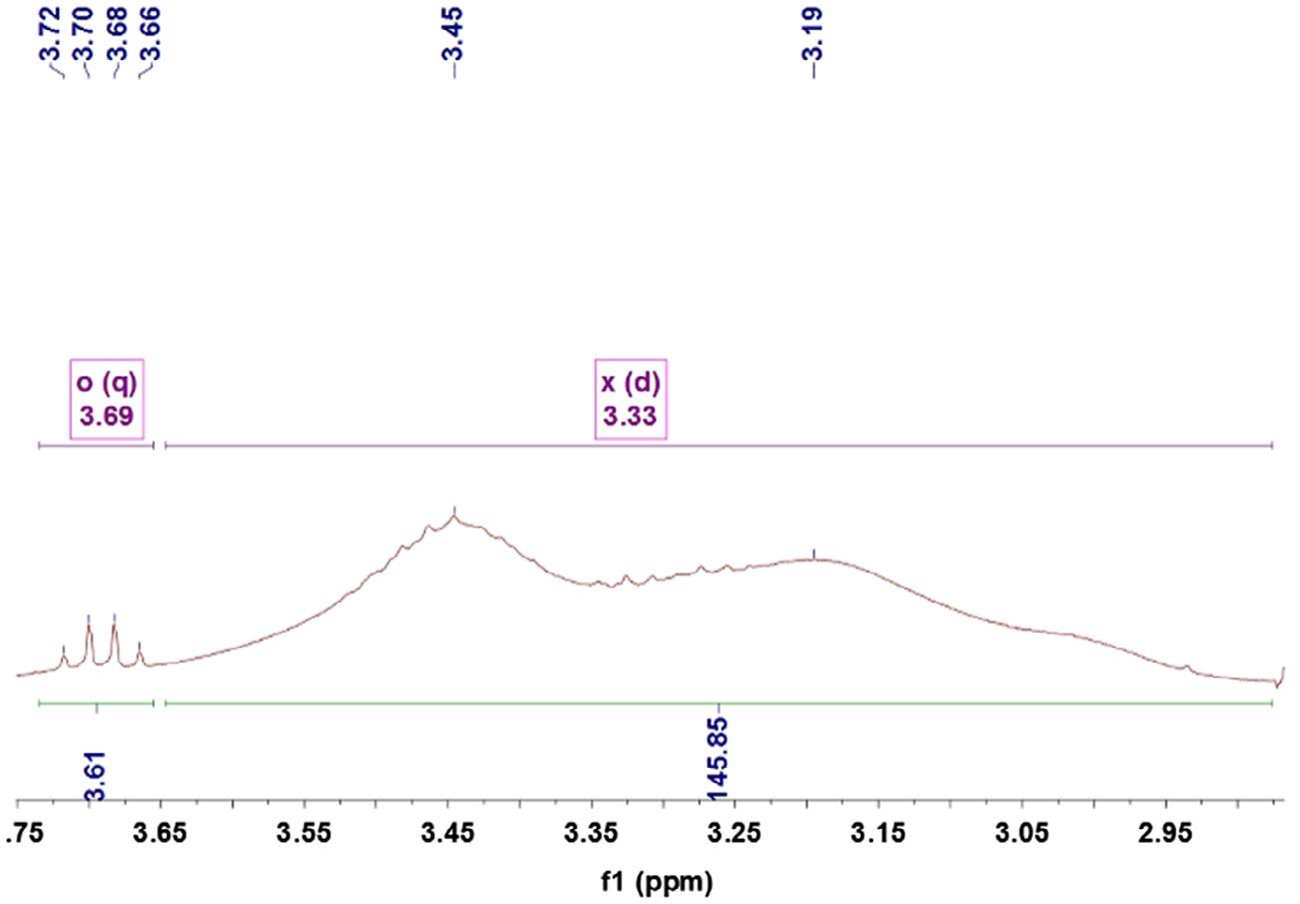
^1^HNMR spectra of the DV3.

**Figure 8.**
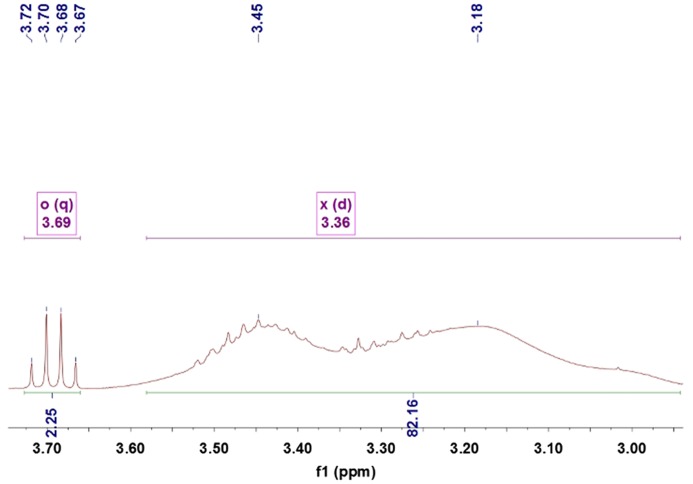
^1^HNMR spectra of the DV4.

**Figure 9.**
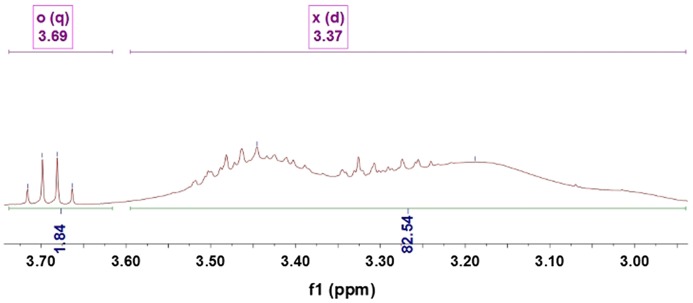
^1^HNMR spectra of the DV5.

Figures 5–9 showed ^1^HNMRspectra of DV, the molar ratio between DEA and NVP segment could also be calculated according to the specific *δ* of DEA and NVP as Equation (2):

(2) $$\frac{n\left(\text{NVP}\right)}{n\left(\text{DEA}\right)}=\frac{\int \text{2H}\left(\delta =3.68\right)}{\int \text{H}\left(\delta =3.31\right)}$$

where, $\int \text{H}\left(\delta =3.68\right)$ and $\int \text{H}\left(\delta =3.31\right)$ represented the total hydrogen quantities of hydrogen at chemical shift 3.68 and 3.31, respectively. There are 2 mol H at *δ*(3.68) in 1 mol NVP, but 4 mol H at *δ*(3.31) in DEA, so as 1 mol NVP combined with 1 mol DEA, the integral of H at *δ*(3.31) would be 2 times that at *δ*(3.68), 127 was the molecular weight of DEA.

As the molar ratio of DEA and NVP was obtained, the molecular weight of PVP could also be calculated using Equation (3):

(3) $${\overset{¯}{M}}_{n}\left(\text{PVP}\right)=\frac{{\overset{¯}{M}}_{n}\left(\text{PDEA}\right)}{M\left(\text{DEA}\right)}\times \frac{n\left(\text{NVP}\right)}{n\left(\text{DEA}\right)}\times M\left(\text{NVP}\right)$$

where, *M*(DEA), *M*(NVP) and ${\overset{¯}{M}}_{n}\left(\text{PDEA}\right)$ represent the molecular weight of DEA, NVP, the molecular weight of PDEA, respectively.

After ${\overset{¯}{M}}_{n}\left(\text{PVP}\right)$ was obtained, the molecular weight of DV could be calculated easily. For example, from Figure 8, it could be found the peak area ratio of x and o was 1: 0.01, so the molecular weight of PDEA could be calculated as Equation (4):

(4) $${\overset{¯}{M}}_{n}\left(\text{DV}1\right)={\overset{¯}{M}}_{n}\left(\text{PDEA}\right)+{\overset{¯}{M}}_{n}\left(\text{PVP}\right)$$

where ${\overset{¯}{M}}_{n}\left(\text{PDEA}\right)$ and ${\overset{¯}{M}}_{n}\left(\text{PVP}\right)$ represent the molecular weight of PDEA and PVP, respectively.

The ^1^HNMR spectra of the DV2, DV3, DV4 and DV5 were shown as Figure 5–9. Similarly, ${\overset{¯}{M}}_{n}\left(\text{DV}2\right)$, ${\overset{¯}{M}}_{n}\left(\text{DV}3\right)$, ${\overset{¯}{M}}_{n}\left(\text{DV}4\right)$, and ${\overset{¯}{M}}_{n}\left(\text{DV}5\right)$ could be obtained as the way of ${\overset{¯}{M}}_{n}\left(\text{DV}1\right)$.

### 3.2. The relationship among the molecular weight, polydispersity index of DV and polymerization time

**Figure 10.**
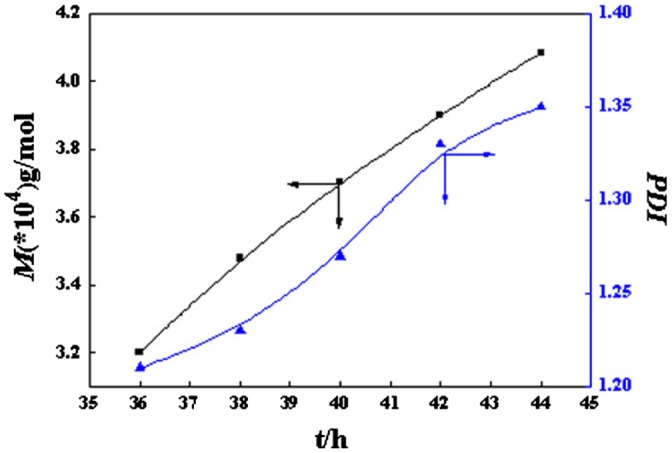
The relationship between relative molecular weight and PDI of PDEA-b-PVP and polymerization time.

Figure 10 displayed the relationships among the molecular weights and poly dispersity index (*PDI*) of DV at different reaction times. It was shown that the molecular weight was linearly increased with the polymerization time, and *PDI* was (1.20, 1.35). The drift off of experimental results to their related theoretical could be ascribed to the variation of viscosity. With prolonged reaction time, the molecular weight of the polymer increased, and the viscosity augmented correspondently [56]. Besides, the concentration of the monomer would decrease with increase of time. Both of which would decrease the polymerization probability, while increase the chain termination probability. As a result, *PDI* became wide gradually with the prolonging of polymerization time.

### 3.3. Thermo-sensitive performance of DV

**Figure 11.**
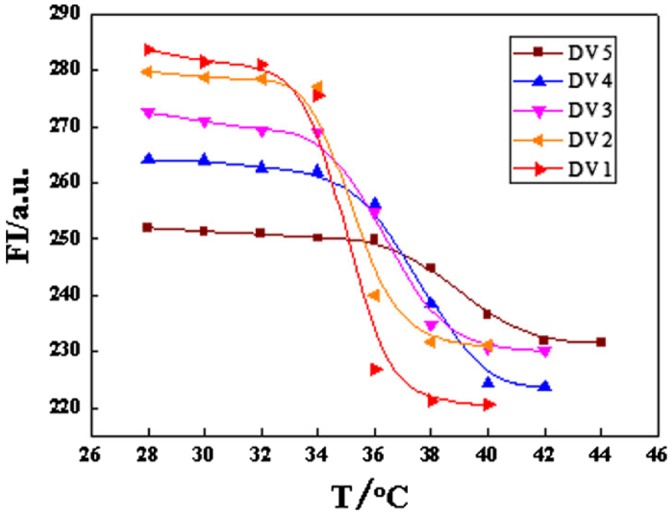
The relationship between fluorescence intensity and temperature of PDEA-b-PVP.

Figure 11 showed the relationship between the molar fraction of DV and LCST in block copolymer. It was shown obviously that the fluorescence intensity (FI) of DV decreased with the increase of the temperature (T). Besides, with the increase of the molar ratio of monomer NVP, the abrupt amplitude of FI was reduced, while LCST increased. Table 1 showed the LCST of DVs.

**Table 1.** The relationship between Mn and LCST.

PDEA-b-PVP	Mn^a^/g⋅mol^−1^	LCST/^o^C
DV1	3413	33.3
DV2	3524	33.8
DV3	3746	34.0
DV4	3857	35.7
DV5	3968	36.0

Notes:

And the molar ratios of NVP to DEA in DV1 to DV5 was 0.038, 0.077, 0.154, 0.192, 0.231, respectively.

a meant the deviation of the Mn of DVs was ±(0.2–0.4)%.

The FI of DV decreased with the increase of temperature, which could be ascribed to the variation of the interaction force between the polymer and solvent. When the temperature was lower than LCST, polymer chains would interact mainly with the solvent by the hydrogen bond and Van der Waals force (VDW) [57]. At this state, solvated molecules would be around macromolecular chains through the hydrogen bond, and a higher ordering solvation shell will form correspondingly, which would lead the polymer chain to an extended random coil [56], the rotation of the molecular chain was relative free, which would be conducive to the fluorescent label, acenaphthylene, absorption energy and emission fluorescence spectrum. As a result, the FI of the polymer was large. As temperature reached LCST, the hydrophobic interaction among polymer chains gradually strengthened, polymer would shrink, and the phase separation would take place slowly, and the rotation of the molecular chain became gradually difficult. At this state, the energy absorption ability of acenaphthylene immobilized on the polymer chain would become difficult, and result in the excitation and fluorescence emission increasing in difficulty, the fluorescence intensity decreased accordingly. As the solvated molecular chain dehydrated, and the hydrophobic part in polymer chains collected together completely. At this stage, the phase separation would finish, and the conformation of polymer chains would change to a stable tight global [58], which would result in the hard rotation of polymer chain. As a result, acenaphthylene immobilized on the polymer chain could not absorb energy and also could not be excited, the fluorescence emission would be less, FI would display a smaller value and keep as a constant, accordingly.

As NVP was introduced, the interaction of the hydrogen bond between NVP and solvent would be strengthened. Thus, the phase separation would become difficult because the process would need to destroy more hydrogen bonds, which would increase LCST of the polymer naturally. With the introduction of NVP, the content of acenaphthylene would decrease the abrupt amplitude of FI reduced naturally.

## 4. Conclusions

This work synthesized DV successfully, and compared the relationship between polymerization time and molecular weight distribution, which verified the synthesis reaction of a block copolymer, which was identified as living polymerization. The thermo-sensitivity study of NVP found that LCST increased with the increase of NVP, while the thermo-sensitivity was weakened, and when the ratio of n_DEA_:n_NVP_ was in the range of (1:0.04, 1:0.05), LCST approximated to 36 °C, which was very close to body temperature.

## Disclosure statement

No potential conflict of interest was reported by the authors.

## Funding

This work was supported by the National Natural Science Foundation of China [grant numbers 51563015, 21762027].
